# A combination nutritional supplement reduces DNA methylation age only in older adults with a raised epigenetic age

**DOI:** 10.1007/s11357-024-01138-8

**Published:** 2024-03-26

**Authors:** Kirsty C. McGee, Jack Sullivan, Jon Hazeldine, Lisa J. Schmunk, Daniel E. Martin-Herranz, Thomas Jackson, Janet M. Lord

**Affiliations:** 1 MRC-Versus Arthritis Centre for Musculoskeletal Ageing Research, Institute of Inflammation and Ageing, University of Birmingham, Birmingham, UK; 2 Hurdle/Chronomics Ltd., London, UK; 3 NIHR Birmingham Biomedcial Research Centre, University Hopsital Birmingham and University of Birmingham, Birmingham, UK

**Keywords:** Inflammation, Inflammaging, DNA methylation, Epigenetic clocks, Nutritional supplement

## Abstract

An increase in systemic inflammation (inflammaging) is one of the hallmarks of aging. Epigenetic (DNA methylation) clocks can quantify the degree of biological aging and this can be reversed by lifestyle and pharmacological intervention. We aimed to investigate whether a multi-component nutritional supplement could reduce systemic inflammation and epigenetic age in healthy older adults.

We recruited 80 healthy older participants (mean age ± SD: 71.85 ± 6.23; males = 31, females = 49). Blood and saliva were obtained pre and post a 12-week course of a multi-component supplement, containing: Vitamin B3, Vitamin C, Vitamin D, Omega 3 fish oils, Resveratrol, Olive fruit phenols and Astaxanthin. Plasma GDF-15 and C-reactive protein (CRP) concentrations were quantified as markers of biological aging and inflammation respectively. DNA methylation was assessed in whole blood and saliva and used to derive epigenetic age using various clock algorithms.

No difference between the epigenetic and chronological ages of participants was observed pre- and post-treatment by the blood-based Horvath or Hannum clocks, or the saliva-based InflammAge clock. However, in those with epigenetic age acceleration of ≥ 2 years at baseline, a significant reduction in epigenetic age (*p* = 0.015) and epigenetic age acceleration (*p* = 0.0058) was observed post-treatment using the saliva-based InflammAge clock. No differences were observed pre- and post-treatment in plasma GDF-15 and CRP, though participants with CRP indicative of an elevated cardiovascular disease risk (hsCRP ≥ 3µg/ml), had a reduction in CRP post-supplementation (*p* = 0.0195).

Our data suggest a possible benefit of combined nutritional supplementation in individuals with an accelerated epigenetic age and inflammaging.

**Supplementary Information:**

The online version contains supplementary material available at 10.1007/s11357-024-01138-8.

## Introduction

In the last decade, a consensus has arisen around the biological processes that drive the aged phenotype, termed the Hallmarks of Aging [1]. The twelve hallmarks consist of primary initiating events which are largely damage based, these then trigger cellular responses such as mitochondrial dysfunction and senescence, which then generate the effector processes [1]. A key effector hallmark is inflammation [1]. As such, the age-related increase in systemic inflammation, inflammaging, is associated with an increased risk of a wide range of chronic conditions including cardiovascular disease, dementia and frailty as well as mortality [2, 3].

A variety of factors contribute to inflammaging, which is characterised by an increase in serum levels of C-reactive protein (CRP) and pro-inflammatory cytokines, Interleukin (IL)-1β, Tumour necrosis factor-alpha (TNF-α), IL-6 and reduction in the levels of anti-inflammatory cytokines such as IL-10 [2, 4]. Factors driving inflammaging include increased adiposity [5], cell senescence [6], microbial dysbiosis [7] and aging of the immune system [8]. The degree of inflammaging could be considered as a biomarker of biological aging [9], indeed machine learning approaches have been used to integrate almost 50 individual inflammatory biomarkers to predict an individual’s inflammatory age, iAGE [10]. Biological age is now considered a much improved indicator of health and mortality risk compared to chronological age [11].

Global biological aging can be measured through specific epigenetic markers, namely DNA methylation at specific genomic sites, termed an epigenetic clock [12–15]. Recently, an epigenetic clock based on DNA methylation marks associated with chronic inflammation, InflammAge, has been developed. This clock also correlates with chronological age and can be measured in saliva [16], increasing its utility in clinical trials or large scale population studies. Importantly, biological age, as indicated by the epigenetic clocks, can be influenced by lifestyle, including diet and exercise [17]. A recent population-based study found an association between dietary patterns with a ‘high inflammatory potential’ and accelerated aging in cardiometabolic disease [18]. This highlights the link between inflammaging and nutrition and the potential for targeting age-associated inflammation and biological/epigenetic age through nutritional supplementation. These interventions could extend healthy lifespan and with economists estimating that extending lifespan by 1 year could save $37 trillion over 10 years, such interventions could be hugely beneficial from both a public health and economic perspective [19].

In this study, we aimed to determine whether a 12-week course of a nutritional supplement containing selected vitamins and nutraceuticals would improve inflammaging and biological age in healthy older adults. The supplement included: Omega 3-polyunsaturated fatty acids and vitamin D to reduce inflammation [20, 21]; vitamin C and an olive derived polyphenol as anti-oxidants; resveratrol [22] and vitamin B3 [23], with the potential to support cellular repair processes; astaxanthin which has both anti-oxidant and anti-inflammatory effects [24]. To assess effects on biological age, we measured DNA methylation, deriving epigenetic clock data from a range of epigenetic clock algorithms as well as the novel saliva-based InflammAge clock. We also considered the impact of the supplement on physical function and quality of life.

## Methods

### Participants and study design

Healthy older adults aged $\ge$ 60 years were invited to take part in the study, based on the inclusion/exclusion criteria outlined in Supplementary Table 1. The study was an uncontrolled, open label interventional study with participants taking a daily multi-component nutritional supplement for 12 weeks. This study duration was selected as several previous studies have shown a reduction in systemic inflammation with nutrients such as fish oils, when supplements were taken for up to 3 months [20]. We were also interested in determining whether a short supplementation period would also be sufficient to modulate epigentic age. Our sample size calculation was based on inflammatory cytokine measurements gained in a previous study by Bayer, using an integrated score from 50 cytokines known to change with age, termed iAGE [10]. The number of participants required at 80% power and a significance level of 0.05 to detect a mean difference of 1.5 years in the iAGE score would be 76. To allow for an approximate 10% drop out we aimed to recruit 85 participants.

Venous blood and saliva samples were collected pre and post supplementation, participants were asked to fast for $\ge 2$ hours prior to blood collection, this was to allow for participants to attend the study centre in the afternoon when an overnight fast would not be practcial. The study was carried out with approval of the UK Health Research Authority and the London-Bromley Research Ethics Committee (Reference: 22/PR/0698). The study was conducted in accordance with the Declaration of Helsinki, with informed and written consent obtained from all participants prior to recruitment into the study. The study is registered with the ISRCTN registry as study ISRCTN77611378.

### Biological sampling

#### Blood sample collection

At baseline (visit 1, V1) and week 12 (visit 2, V2), peripheral venous blood samples were acquired by venepuncture into vacutainers (BD Biosciences, Oxford, UK) containing ethylenediaminetetraacetic acid (EDTA) or lithium heparin (LH). Plasma was isolated from blood collected in vacutainers containing LH or EDTA by centrifugation at 461 × *g* for 8 min at room temperature. Plasma was stored at -80°C.

#### Saliva sample collection

Saliva samples were collected at baseline and week 12 for downstream DNA Methylation analysis using Hurdle’s saliva sample collection kit (ORAgene DNA Saliva Collection kit; OG-600; DNA Genotek, UK) and stored at 4°C prior to analysis.

#### Nutraceutical supplement composition

The daily dose of the supplement, supplied by Bayer Consumer Care AG, contained Vitamin D3 (20 µg), Vitamin B3 (niacinamide, 50 mg), Vitamin C (85 mg), Omega-3 polyunsaturated fatty acids (eicosapentaenoic acid (EPA) and docosahexaenoic (DHA) acid, 250 mg), olive fruit extract (delivering 10 mg hydroxytyrosol), resveratrol (30 mg) and astaxanthin (3.2 mg). Participants were required to cease consumption of any multivitamins, dietary supplements containing (or any food/beverage products supplemented with) any of the study nutraceutical components, at least three weeks prior to commencement of the study.

#### Physical function and quality of life assessment

Physical function was assessed using the short physical performance battery (SPPB) [25]. Briefly, participants were asked to sit to stand from a chair (time taken for 5 rises), perform a balance test and do a timed 4-m walk speed test, all performed at baseline (pre-intervention) and at week 12. The maximum score in the SPPB test is 12 (10 or above indicates robust health and 3–9 indicates frailty).

Quality of life (QoL) was assessed using the SF36 questionnaire [26], completed at baseline, and 4, 8, and 12 weeks into the intervention. The SF36 gives an overall score for QoL and covers 9 different domains, each with a maximum possible score of 100.

#### DNA methylation (DNAm) analysis

Bisulphite conversion and array processing was performed by Eurofins Genomics, Denmark, using the Infinium 850K EPIC array (Illumina, CA, USA). All data processing for Horvath, Hannum, DNAm PhenoAge and GrimAge calculations was performed using RStudio (Version: 2023.06.1 + 524). IDAT files were processed using minfi [27] and ENMIX [28]. Participant data were excluded if one or both of their samples did not pass ENMIX quality control. ENMIX, “RELIC” dye bias correction and quantile-normalisation was performed, low-quality and outlier probe values were excluded. All data processing and QC for InflammAge was calculated as previously described [16].

#### Epigenetic age estimation

Horvath [12], Hannum [13] and DNAm PhenoAge [14], DNA methylation age analysis was performed using the ENMIX package. GrimAge [15] was calculated using the dnaMethyAge package [29]. The mean of the Horvath, Hannum, DNAm PhenoAge and GrimAge epigenetic ages was generated for each participant and presented as a value called “Mean EpiAge”. Mean EpiAge analysis was included to accommodate differences between DNAm age and chronological age gained with the different clocks [30]. To adjust for chronological age as a confounding factor, epigenetic age acceleration was defined as the residual of a linear regression of the epigenetic age on chronological age. InflammAge and InflammAge acceleration were calculated as previously described [16].

#### Serum protein analysis

Concentrations of plasma GDF-15 were quantified using a commercially available ELISA kit (DY957, Bio-techne, USA) following the manufacturer’s guidelines. Plasma CRP concentrations were measured using a commercially available ELISA kit (EU59151, IBL International, Germany), following the manufacturer’s guidelines.

### Statistical analyses

Data analysis was carried out using the GraphPad Prism® software package v9 (GraphPad Software Ltd, California, USA). Epigenetic data analysis was performed using R Studio (Version: 2023.06.1 + 524). Normality of data was assessed using the Shapiro–Wilk test. For normally distributed data, statistical analysis was carried out using either student *t-*test or a paired student *t-*test, whilst non-parametric data were analysed using an unpaired Wilcoxon or Wilcoxon matched-pairs signed rank test. The Spearman test was used to assess correlation. The threshold for statistical significance was set at *p* < 0.05.

## Results

### Participant demographics

Venous blood and saliva samples were obtained from 83 participants (age 72.2 ± 6.4 years; BMI: 26.6 ± 3.99 kg/m^2^; 31 males, 52 females) at baseline (pre-intervention) and from 80 participants (71.85 ± 6.23 years; BMI: 25.75 ± 3.94; 31 males, 49 females) 12 weeks post nutritional supplementation. Participant characteristics for those who completed the study are shown in Table 1. Compliance was determined by counting tablets returned at the end of the study and was found to be 100%. A Consort diagram for the study is shown as Supplementary Fig. 1.

**Table 1:** Participant characteristics

	Age in years	BMI (Kg/m^2^)	Ethnicity
	(mean ± SD)	(mean ± SD)	(white:non-white)
Total (n = 80)	71.85 ± 6.23	25.75 ± 3.94	75:5
Male (n = 31)	73.90 ± 5.41	26.26 ± 3.02	29:2
Female (n = 49)	70.55 ± 6.41	25.42 ± 4.42	46:3

### Effect of a nutraceutical supplement on inflammation

Our first aim was to investigate the potential influence of the combined nutraceutical supplement on biomarkers associated with age-related systemic inflammation. For the whole cohort, CRP levels showed no significant change following the supplement intervention, mean CRP at V1 = 1.51 ± 2.43 µg/ml and V2 = 1.65 ± 2.44µg/ml (*p* = 0.546). However, a subgroup analysis categorising CRP levels according to risk of cardiovascular disease: < 1µg/ml (low risk), 1–2.9µg/ml (moderate risk), and ≥ 3µg/ml (higher risk) [31, 32], revealed a significant 28.3% decline in CRP levels following treatment in the nine participants in the high-risk group compared with V1 (*p* < 0.0195; Table 2).

**Table 2:** The effect of supplement on plasma GDF-15 and CRP

	Visit 1 (Baseline)	Visit 2 (Week 12)	*p*-value
GDF-15 (pg/ml)	237 ± 84.9, n = 83	239 ± 88.6, n = 80	0.672
CRP ≤ 1 µg/mL	0.43 ± 0.27, n = 52	1.04 ± 2.22, n = 50	0.106
CRP 1–2.9 µg/mL	1.71 ± 0.57, n = 22	1.56 ± 0.78, n = 21	0.303
CRP ≥ 3 µg/mL	7.28 ± 3.7, n = 9	5.22 ± 3.27, n = 9	**0.0195**

**Fig. 1:**
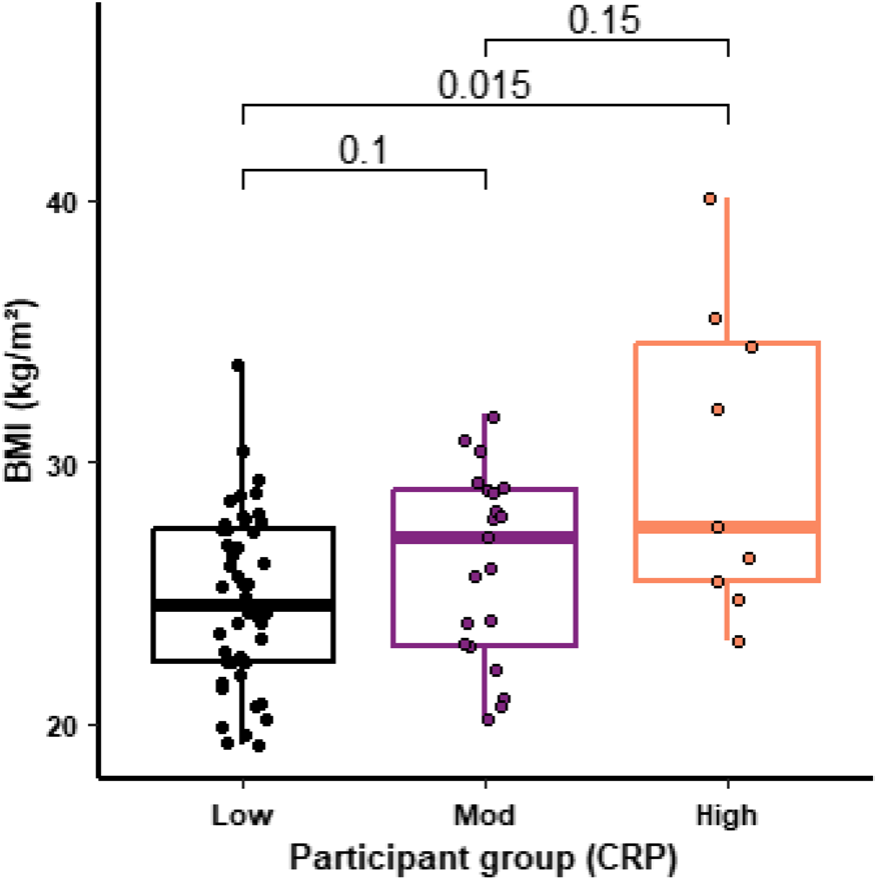
CRP levels and BMI. The figure shows the BMI for study participants with a CRP indicative of Low (< 1.0 µg/ml; *n* = 52), Moderate (1–2.9 µg/ml; *n* = 22) or High (≥ 3.0 µg/ml; *n* = 9) risk of cardiovascular disease. For each box plot the central line is median, the bottom line is the 1st quartile (Q1), the top is the 3rd quartile (Q3). The whiskers represent calculated minimum and maximum values using the interquartile range (IQR). *P* values are shown above each compared dataset

The nine participants in the higher CVD risk group exhibited a significantly higher BMI when compared to the low-risk group (*p* < 0.015; Fig. 1). No significant effect of the supplement was observed for GDF-15 (Table 2), a marker linked to aging [33].

### Influence of nutraceutical treatment on blood epigenetic age

We then sought to assess the impact of the nutraceutical supplement on biological age by assessing epigenetic age. After quality control (QC) analysis of the DNA methylation data, samples of 79 participants were included (median age at baseline: 72.42 years, range: 60.27–87.71; median BMI at baseline: 25.77 kg/m^2^, range: 19.2–40.1; 31 males, 48 females).

**Fig. 2:**
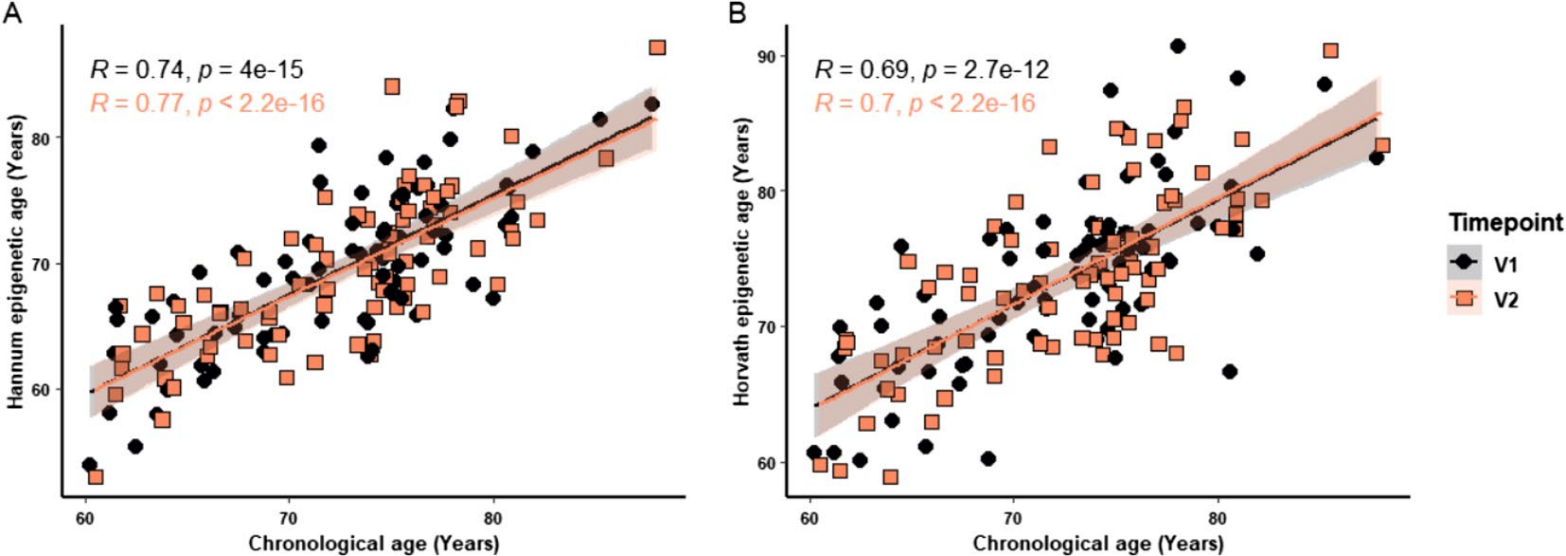
Correlation of chronological age with DNA methylation age. The Hannum (**A**) and Horvath (**B**) epigenetic ages were correlated with chronological age for combined data from visit 1 (circles) and visit 2 (squares). Hannum: *n* = 79; V1: *p* < 0.0001, *R* = 0.74; V2: *p* < 0.0001, *R* = 0.77. Horvath: *n* = 79; V1: *p* < 0.0001, *R* = 0.69; V2: *p* < 0.0001, *R* = 0.70. Correlations were assessed using the Spearman test. P values are shown above each compared dataset

We found a highly significant correlation between chronological age and epigenetic age for both baseline (V1) and week 12 (V2) calculated using the Hannum (V1: *p* < 0.0001, *R* = 0.74; V2: *p* < 0.0001, *R* = 0.77; Fig. 2A) and Horvath (V1: *p* < 0.0001, *R* = 0.69; V2: *p* < 0.0001, *R* = 0.70; Fig. 2B) clocks.

**Fig. 3:**
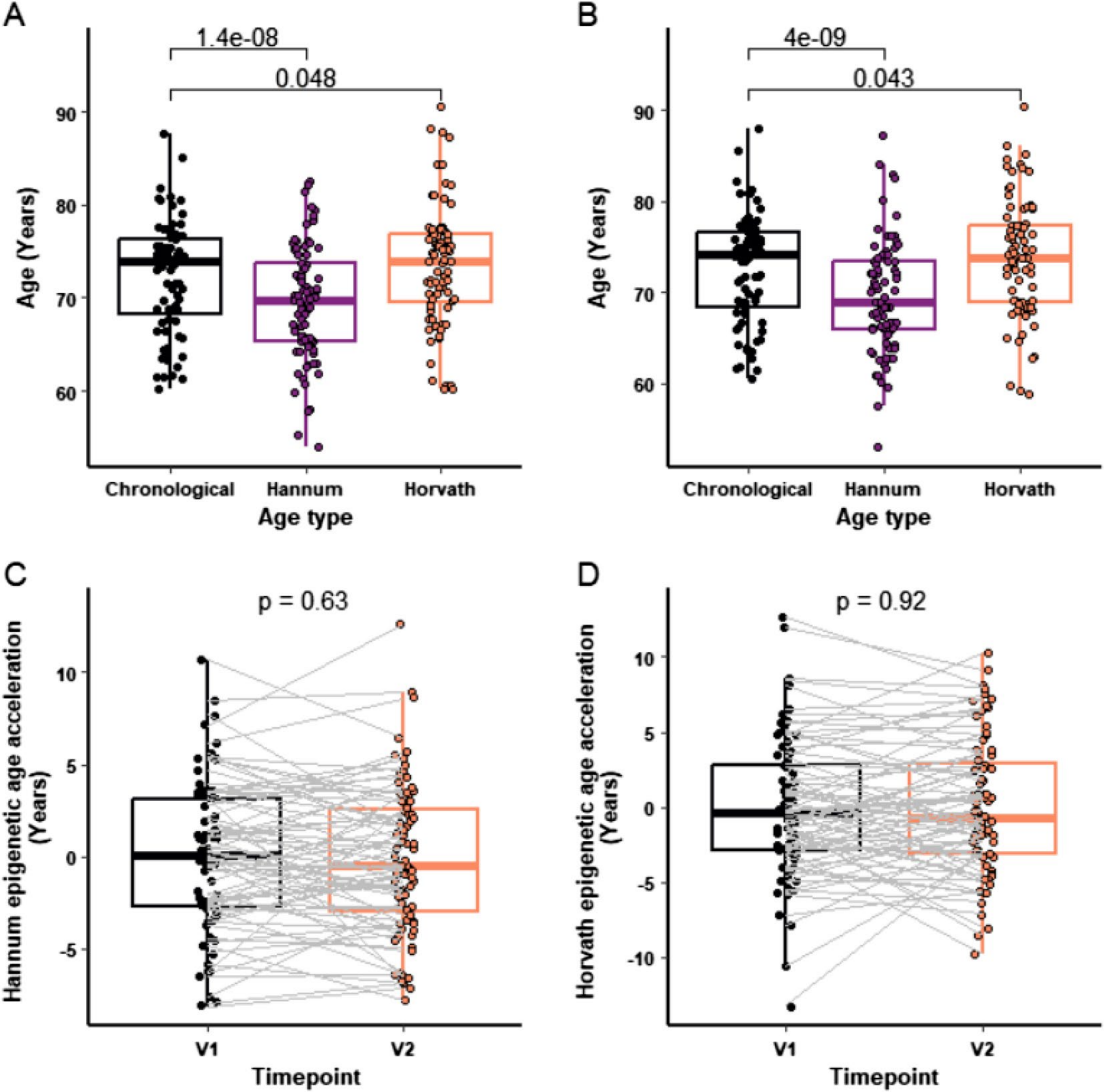
Epigenetic age analysis pre and post supplement. Hannum and Horvath epigenetic ages were compared to chronological age at baseline, V1 (**A**) and post supplement, V2 (**B**). The Hannum (**C**) and Horvath (**D**) epigenetic ages of participants were compared between V1 and V2. For each box plot the central line is median, the bottom line is the 1st quartile (Q1), the top is the 3rd quartile (Q3). The whiskers represent calculated minimum and maximum values using the interquartile range (IQR). Statistical analysis was carried out using paired Student *t-*test (*n* = 79). *P* values are shown above each compared dataset

For the epigenetic age analysis, we assessed changes in epigenetic age but also effects on the difference between epigenetic age and chronological age, or epigenetic age acceleration, as this difference is associated with clinical outcome [34]. Hannum and Horvath epigenetic age were derived, both before (Fig. 3A) and after (Fig. 3B) nutritional supplementation. Epigenetic age was higher than chronological age, using the Horvath clock, at both visit 1 (Fig. 3A; *p* = 0.048) and visit 2 (Fig. 3B; *p* = 0.043), but there was a significantly lower Hannum epigenetic age compared to chronological age at visit 1 (Fig. 3A; *p* < 0.0001) and visit 2 (Fig. 3B; *p* < 0.0001). Further analysis examining the difference in Hannum and Horvath epigenetic age acceleration of participants pre and post nutraceutical treatment revealed no significant change in participant epigenetic age acceleration following treatment, for either clock (Fig. 3C and D, respectively).

### Epigenetic age across four different clocks

**Fig. 4:**
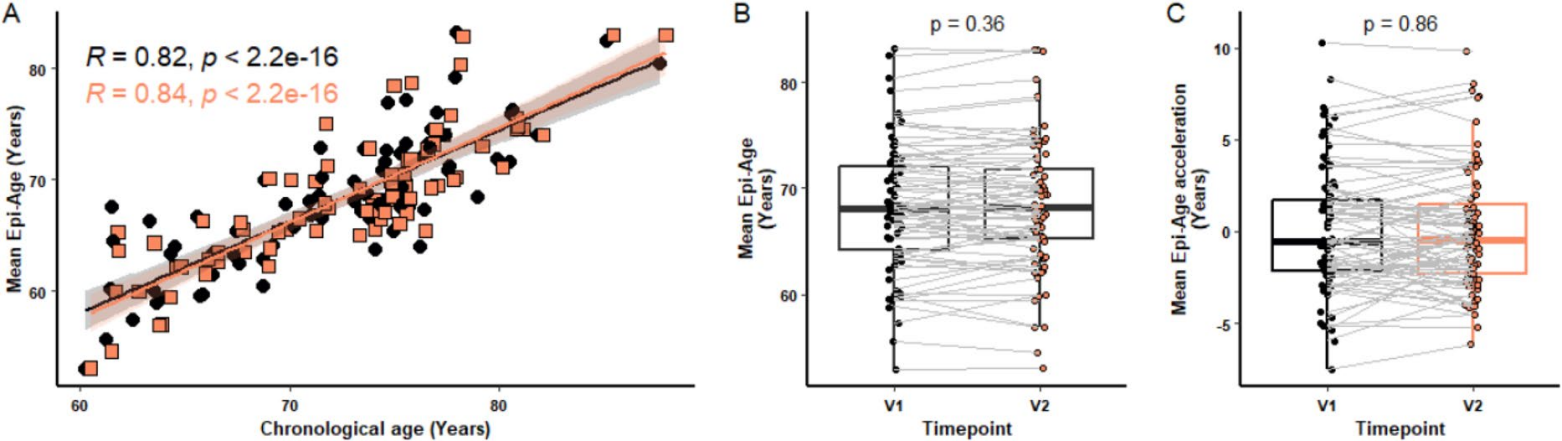
Mean of epigenetic age across four clocks. GrimAge, Horvath, Hannum and PhenoAge clocks were calculated and a Mean Epi-Age score produced (*n* = 79). The figure shows correlation between mean epi-age and chronological age (**A**), the difference in mean epi-age (**B**), and acceleration in Mean-Epi-Age (**C**) between visits 1 and 2. For each box plot the central line is median, the bottom line is the 1st quartile (Q1), the top is the 3rd quartile (Q3). The whiskers represent calculated minimum and maximum values using the interquartile range (IQR). *P* values are shown above each compared dataset. The following statistical analysis was carried out: (**A**) Spearman test, (**B**) paired Student *t-*test, and (**C**) paired student *t-*test and Wilcoxon matched-pairs signed rank test

Fahy et al. [30] assessed epigenetic age by averaging the data from four different epigenetic clocks (Hannum, Horvath, DNAmPhenoAge and GrimAge) to provide a composite ‘mean epi-age’. As we observed some variation in the calculated epigenetic age depending on the clock being used, we applied this method after also calculating epigenetic age using the GrimAge [15] and DNAmPhenoAge [14] clocks. As can be seen in Fig. 4A, a strong correlation between the mean epi-age scores and chronological age was seen, validating this composite ‘mean epi-age’ model (*p* < 0.0001). Further analysis looking at the potential effect of nutraceutical treatment on the mean epi-age (Fig. 4B) and mean epi-age acceleration (Fig. 4C) showed there to be no significant difference between visits 1 and 2.

### Influence of baseline epigenetic age on response to a nutraceutical supplement

**Fig. 5:**
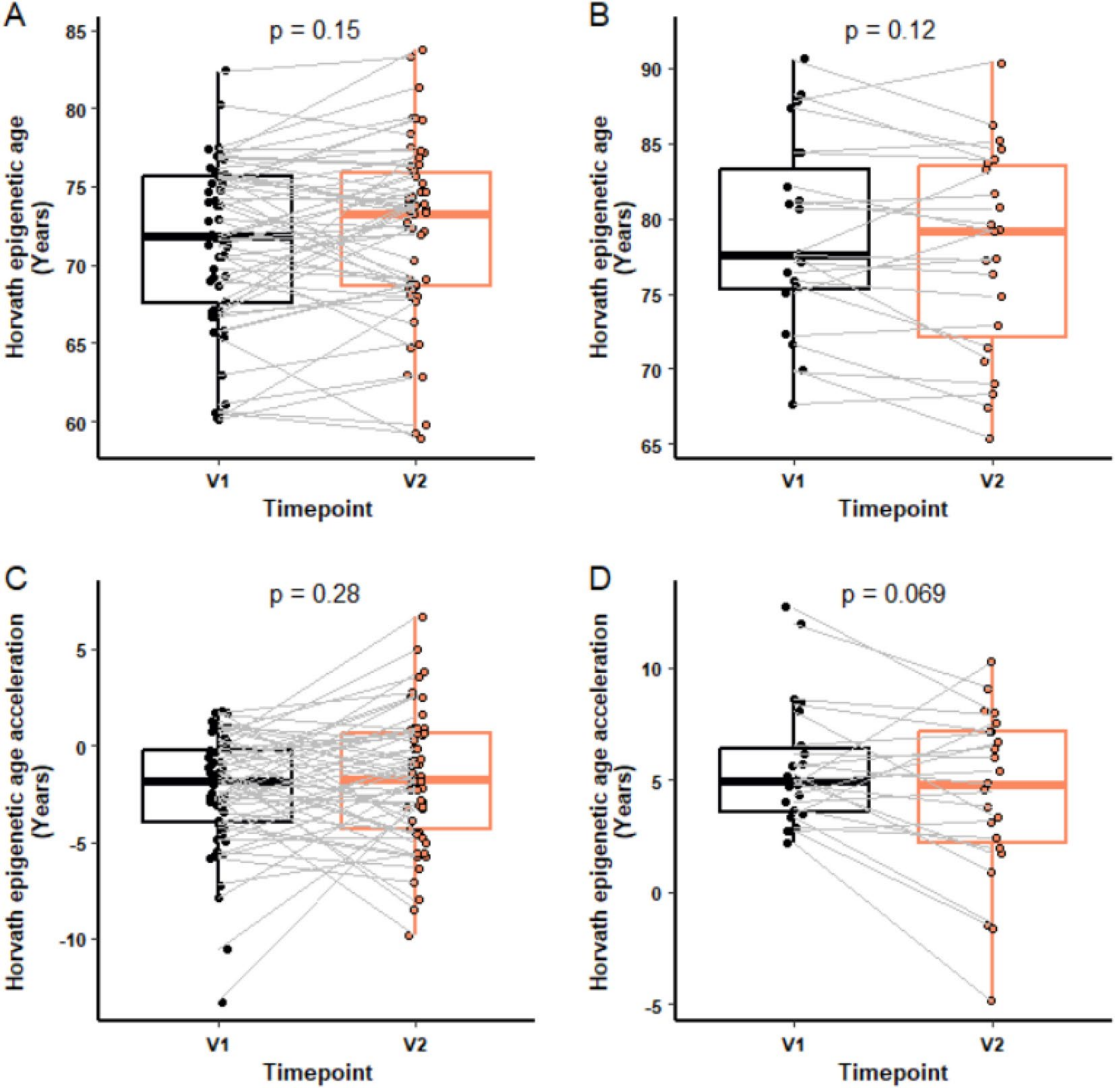
Horvath epigenetic age acceleration by baseline age acceleration. Epigenetic age and epigenetic age acceleration at V1 and V2 was compared in participants who had a Horvath epigenetic age acceleration under 2 years (*n* = 56) or over 2 years (*n* = 23) 2 at V1 (baseline). Horvath epigenetic age at V1 and V2 in those without (**A**) or with (**B**) Horvath age acceleration over 2 years at baseline. Horvath epigenetic age acceleration at V1 and V2 in participants without (**C**) or with (**D**) Horvath epigenetic age acceleration over 2 years at baseline. For each box plot the central line is median, the bottom line is the 1st quartile (Q1), the top is the 3rd quartile (Q3). The whiskers represent calculated minimum and maximum values using the interquartile range (IQR). *p*-values are shown above each compared dataset. The following statistical analysis was carried out: (**A**) Wilcoxon matched-pairs signed rank test, (**B**–**D**) paired Student *t-*test

To investigate whether the baseline epigenetic age of a participant had any influence on the participant’s response to the intervention, we examined the difference in epigenetic age and epigenetic age acceleration, using the Horvath clock, following supplementation in those whose epigenetic age was greater than their chronological age by 2 years or more. No significant difference in epigenetic age was found between V1 and V2 in participants with less than 2 years of Horvath epigenetic age acceleration at baseline (*n* = 56; Fig. 5A) or more than 2 years (*n* = 23; Fig. 5B). However, for those with epigenetic age acceleration at baseline, there was a non-significant trend of a 21.5% decline, equivalent to 1.98 years, in epigenetic age acceleration following supplement treatment (*p* < 0.069; Fig. 5D), with no effect on those with no acceleration at baseline (Fig. 5C).

### Influence of nutraceutical treatment on epigenetic age using the saliva-based InflammAge clock

**Fig. 6:**
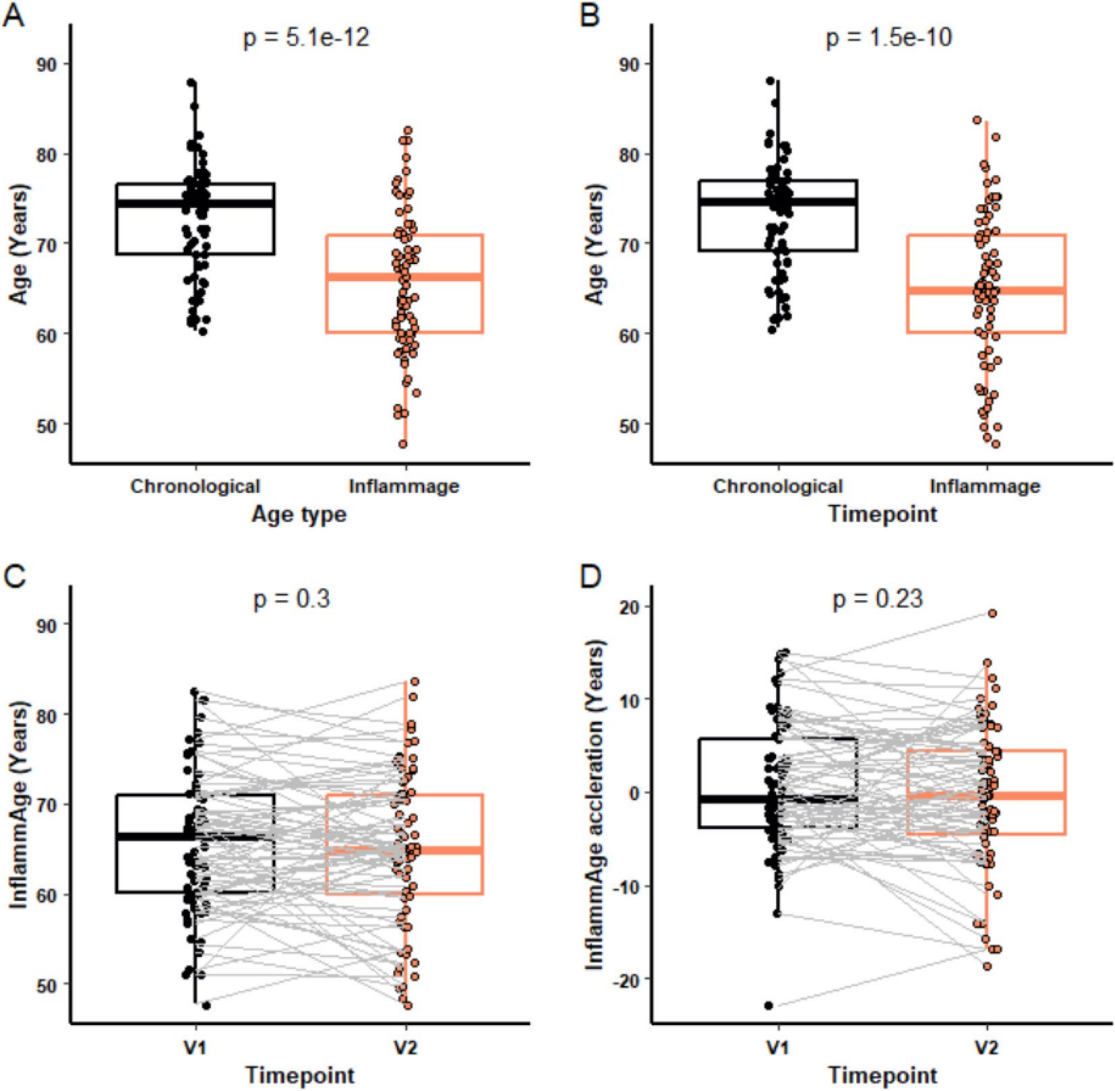
InflammAge analysis pre and post supplement. InflammAge was compared to chronological age at baseline, V1 (**A**) and post supplement, V2 (**B**). InflammAge (**C**) and InflammAge acceleration (**D**) of participants was compared between V1 and V2. For each box plot the central line is median, the bottom line is the 1st quartile (Q1), the top is the 3rd quartile (Q3). The whiskers represent calculated minimum and maximum values using the interquartile range (IQR). *P* values are shown above each compared dataset (*n* = 75). The following statistical analysis was carried out: (**A**) paired Student *t-*test, (**B**) Wilcoxon matched-pairs signed rank test, (**C**–**D**) paired Student *t-*test

In addition to the established epigenetic clocks of Hannum and Horvath, we also used a novel algorithm (InflammAge) that gives an estimated DNA methylation (biological) age for saliva samples and was trained on chronic inflammation markers [16]. Following initial QC analysis, 75 participant samples were analysed (mean age at baseline 72.77 years (60.31–87.78); 28 males, 47 females). Using this analysis, we found a significantly lower InflammAge than chronological age at both V1 (*p* < 0.0001, Fig. 6A) and V2 (*p* < 0.0001, Fig. 6B) using this clock, but there was no change in the InflammAge (*p* = 0.3, Fig. 6C) or the degree of InflammAge acceleration (*p* = 0.23; Fig. 6D) of participants following supplement treatment.

### Effect of baseline InflammAge on response to a nutraceutical supplement

**Fig. 7:**
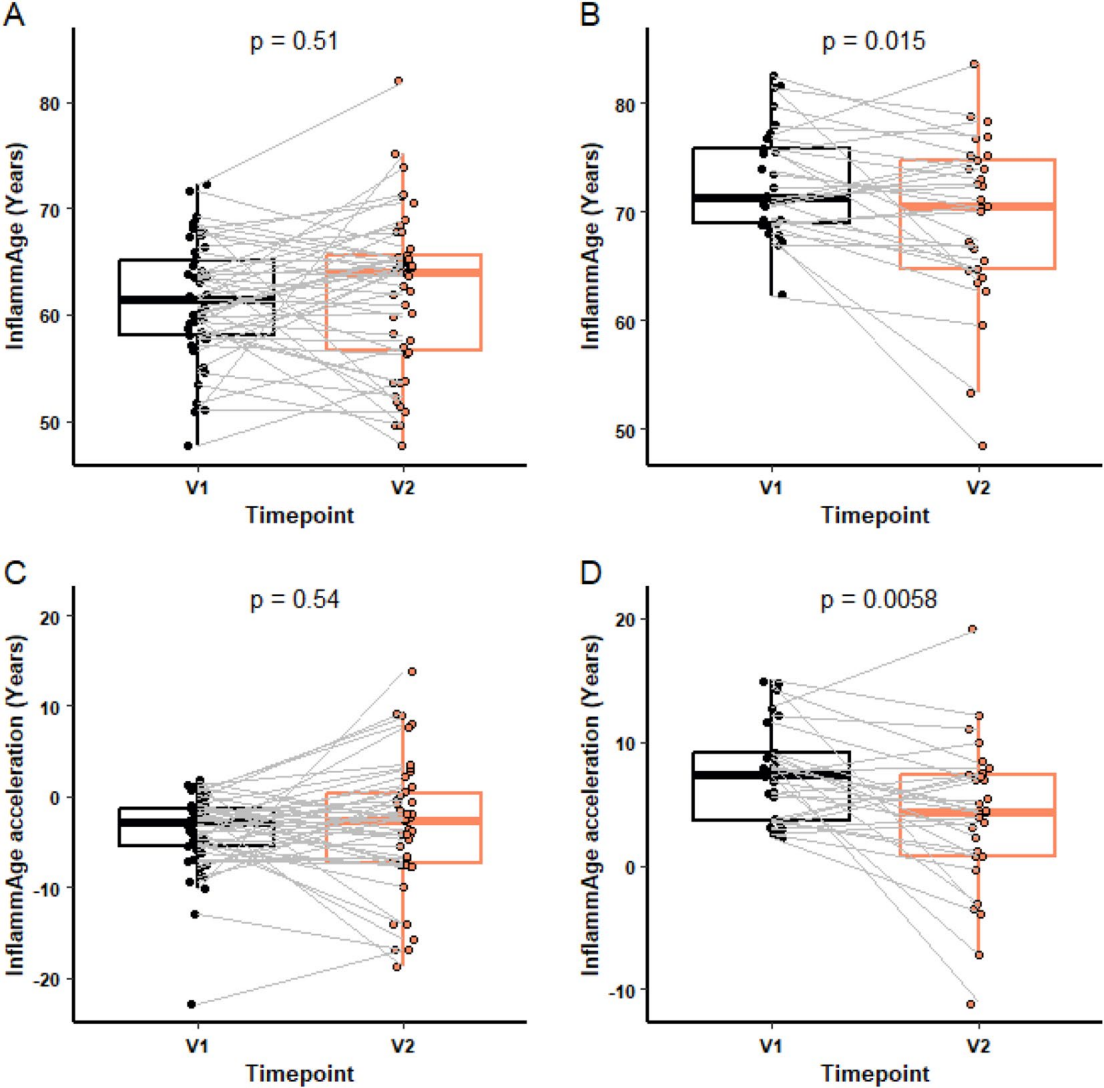
InflammAge analysis by baseline age pre and post supplement. InflammAge at V1 and V2 was compared in participants (**A**, *n* = 46) with InflammAge acceleration < 2 years or (**B**, *n* = 29) with InflammAge acceleration ≥ 2 years at V1 (baseline). InflammAge acceleration at V1 and V2 was compared in participants without (**C**, *n* = 46) or with (**D**, *n* = 29) InflammAge acceleration ≥ 2 years at baseline. For each box plot, the central line is median, the bottom line is the 1st quartile (Q1), the top is the 3rd quartile (Q3). The whiskers represent calculated minimum and maximum values using the interquartile range (IQR). *P* values are shown above each compared dataset. The following statistical analysis was carried out: (A + D) paired Student *t-*test, (B + C) Wilcoxon matched-pairs signed rank test

As with the Horvath clock, we wanted to determine if an individual’s baseline InflammAge influenced the response to the supplement. 29 participants had a baseline InflammAge of ≥ 2 years higher than their chronological age. The supplement reduced the InflammAge score in this group by 4.055%, equivalent to 3.31 years, (*p* = 0.015; Fig. 7B) and the degree of InflammAge acceleration by 46.77%, equivalent to 3.47 years, (*p* = 0.0058; Fig. 7D). No difference was observed in participants with no InflammAge acceleration at baseline (Fig. 7A and C).

### Sex differences in blood and saliva epigenetic age following nutraceutical treatment

Given established sex differences in the rate of biological aging [35], we sought to investigate potential sex-based variations in epigenetic age response to the supplement. As can be seen in Supplementary Fig. 2, no significant change in epigenetic age acceleration was seen following supplement treatment with either the Horvath (Suppl. Figure 2A and 2B) or Hannum (Suppl. Figure 2C and 2D) clocks when stratifying for females and males. There was a strong trend to a decrease in epigenetic age acceleration in female participants with the novel InflammAge clock (*p* = 0.05, Suppl. Figure 2E), while no difference was observed in males (*p* = 0.8, Suppl. Figure 2F).

### Physical function and quality of life assessments

The ability of the nutraceutical supplement to improve features of physical functioning and quality of life (QoL) were also assessed using the Short Physical Performance Battery (SPPB) and SF36 questionnaire respectively. Participants were in very robust health overall, indicated by a median score of 12 in the SPPB test and there was no change in the mean SPPB score of 11.4 following treatment (Table 3). Participants with the lowest quartile score (mean 10.1) showed a non-significant increase to 10.8 following treatment (*p* = 0.14).

**Table 3:** The effect of supplement on SPPB and SF36

All					Low at baseline			
	Median	Mean	Baseline	12 wks		Baseline	12wks	
SPPB	12 (1)	11.4 (1.02)	11.4 (1.2)	11.4 (0.9)	*Z* = -1.7 *p* = 0.09	10.1 (1.4)	10.8 (1.0)	*Z* = -1.5 *p* = 0.14
SF-36 (total)	751 (118)	725 (103)	730 (90.8)	721 (114.9)	*Z* = -0.2 *p* = 0.84	626 (70.5)	663 (137.4)	*Z* = -1.73 *p* = 0.08
Physical function	95 (15)	90.1 (11.5)	89.7 (12.3)	90.7 (11.0)				
Physical health	100 (0)	84.6 (31.5)	85.8 (30.4)	82.2 (34.3)				
Emotional problems	100 (0)	95.7 (15.7)	96.1 (15.0)	95.0 (18.4)				
Energy	70 (30)	68.1 (19.8)	68.6 (19.6)	68.8 (20.4)				
Emotional score	88 (12)	85.4 (10.9)	84.7 (11.6)	84.2 (13.1)				
Social	100 (12.5)	90.0 (20.1)	89.4 (20.8)	88.3 (23.9)				
Pain	90 (30)	82.0 (18.3)	82.2 (18.7)	81.5 (20.2)				
General health	80 (70)	77.3 (15.1)	76.88 (15.0)	76.6 (14.5)				
Health change	50 (0)	52.5 (14.5)	51.83 (14.2)	54.1 (15.1)				

The supplement had no effect on the overall score for the nine QoL domains tested (median SF36 score: 751; Table 3). Participants with the lowest quartile mean SF36 score showed a trend towards a positive effect of the supplement (V1: 626, V2: 663, *p* = 0.08).

## Discussion

The systemic low-grade inflammation associated with advancing age, inflammaging, is a risk factor for many age-related diseases and mortality [2] and has been recently added to the Hallmarks of Aging as a driver of the aging process [1]. As it is well established that diet influences both inflammation and healthspan [36], and that this influence includes epigenetic modifications [37], we sought to explore the impact of a 12-week nutritional supplement on inflammation and indicators of biological aging. For biological age, we used DNA methylation data to derive calculations for epigenetic clocks, including a novel saliva-based epigenetic clock trained using a set of DNA methylation markers predictive of chronic inflammatory protein levels, InflammAge [16]. Overall, we found that 12-weeks of the multi-component nutritional supplement had a limited effect on inflammation and epigenetic markers of biological age in healthy older adults. Although in the cohort as a whole, we saw no effects on inflammation or epigenetic age, we did see effects in specific sub-groups. Those with a CRP level considered at higher risk of cardiovascular disease, ≥ 3µg/ml [31], had reduced CRP after the treatment period. In participants whose epigenetic age was higher than their chronological age, there was a reduced epigenetic age and epigenetic age acceleration using the InflammAge clock and a trend towards an effect with the blood-based Horvath clock. A slight beneficial effect (*p* = 0.05) of the supplement was seen on epigenetic age acceleration, calculated using the InflammAge clock, in females irrespective of the baseline epigenetic age. We conclude that the supplement is effective in those on an unhealthy aging trajectory.

The observation of a reduced CRP in the subgroup of participants with elevated baseline CRP (≥ 3µg/ml, *n* = 9) suggested utility in those with raised inflammation status. A 12-week open label combination nutraceutical intervention study in individuals with elevated CRP (> 2µg/mL) and low-density lipoprotein (LDL) cholesterol of 100–160 mg/dL also found a significant reduction following treatment [38]. In that study, the nutraceuticals were selected for their cholesterol lowering properties and some such as astaxanthin for their anti-inflammatory and anti-oxidant effects [24]. We did not measure cholesterol or blood lipids in our study. A recent randomised case control study comparing the effects of a combined nutraceutical (including vitamins C, D and E) in young (18–50 years) and hospitalised older (> 65 years) participants found a significant reduction in circulating IL-6 and CRP levels in older participants following 12-week treatment [39]. Taken together, these findings support the contention that these combined nutraceutical supplements will be most effective in those either already not in robust health, or at risk of age-related diseases such as cardiovascular disease.

This conclusion is also supported by our findings for markers of biological aging, namely epigenetic clocks. A significant reduction in InflammAge and InflammAge acceleration was observed post supplement in the twenty-nine participants who had a raised InflammAge score at baseline. We did not find a significant effect with the blood-based clocks, though there was a trend towards a reduction with the Horvath clock. This difference in outcome may reflect the fact that the InflammAge clock was built to quantify chronic inflammation and the nutritional supplement contains nutrients with known anti-inflammatory effects, such as Omega 3-PUFAs [20] and astaxanthin [24]. There are few interventional studies that have used nutrition to modulate epigenetic clocks. Observational data using the InChianti and Womens Health Initiative datasets has shown a relationship between fish, poultry and fruit and vegetable intake and lower epigenetic age [17]. A randomised controlled study of epigenetic age was carried out in 43 older males (50–72 years) undertaking an 8-week programme of lifestyle guidance, supplemented with probiotics and phytonutrients. The nutritional programme was complex but primarily used agents known to target DNA methylation processes, for example α-ketoglutarate which modulates methylation biosynthetic pathways [40] and curcumin and quercetin which target DNA methyltransferases [41]. The intervention achieved a non-significant (*p* = 0.066) reduction in DNA methylation age of 1.96 years using the Horvath clock [42]. Further large scale, longer studies are clearly needed to establish whether epigenetic age can be reduced by simple nutritional supplements, if so which ones and importantly do the changes actually associate with improved healthspan.

Our study has some limitations, primarily that it was an open label, uncontrolled study, future studies with the supplement should be blinded, include a placebo. It is also important to point out that the study sample size was based on reducing age-related systemic inflammation rather than DNA methylation age. Using the blood-based Horvath data for those participants with an epigenetic age > 2 years above their chronological age, we estimate that a sample size of 34 per group would be required to demonstrate a 2-year difference in epigenetic age acceleration. As we had only 23 participants with an epigenetic age greater than 2 years, we were slightly underpowered, though the InflammAge clock was able to detect changes in a similar sample size (*n* = 29). In addition we did not assess serum levels of the nutrients before and after supplementation, variability in diet may therefore have influenced circulating levels of nutrients such as polyunsaturated fatty acids. It is also a consideration that our group were all in robust health with only nine of the participants having a raised CRP. Future studies should focus on a cohort with inflammaging or a raised epigenetic age. Such a design has been proposed recently for an intervention with α-ketoglutarate [43].

It is also notable that many studies that have reported a decrease in epigenetic age have been conducted for a longer period than the present study. For example, a trial using a cocktail of metformin, DHEA and growth hormone, lasted 12 months [30]. Demidenko et al. found that following an average 7-month use of rejuvant©, a supplement containing α-ketoglutarate and vitamins, led to a mean reduction in biological age of 8 years, measured using the saliva-based DNAm clock, TruAge [44]. Furthermore, many shorter nutritional intervention studies that do show a significant shift in biological age involve cohorts with a morbidity associated with age acceleration, e.g. obesity [45]. Therefore our exclusion criteria may have eliminated those adults most lieklyt o benefit from the intervention.

## Conclusion

Taken together, although the effect was modest, and only seen with the InflammAge clock, these data are promising as reducing a biological age measure in 12 weeks should be seen as a positive indicator for future trials. Crucially, the data suggest that nutritional supplements may be best targeted at those on a negative aging trajectory, i.e. with signs of inflammaging or a raised epigenetic age.

### Supplementary Information

Below is the link to the electronic supplementary material.

Supplementary file1 Supplementary Figure 1. Conosrt diagram for recruitment to the study. (DOC 27 KB)

Supplementary file2 Supplementary Fig 2: Effect of sex on epigenetic age response to supplement. The degree of epigenetic age acceleration in individuals at V1 and V2 were compared by the Horvath clock for females (A, n=48) and males (B, n=31), by the Hannum clock for females (C, n=48) and males (D, n=31), and by the InflammAge clock for females (E, n=47) and males (F, n=28). For each box plot the central line is median, the bottom line is the 1st quartile (Q1), the top is the 3rd quartile (Q3). The whiskers represent calculated minimum and maximum values using the interquartile range (IQR). P values are shown above each compared dataset. Statistical analysis was carried out using paired Student t-test. (PDF 165 KB)

Supplementary file3 (DOCX 21 KB)

## Data Availability

The original data generated in the study will be made available following a reasonable request to the corresponding author.
