# Supplementary material for: A combination nutritional supplement reduces DNA methylation age only in older adults with a raised epigenetic age
Source: GeroScience. 2024 Mar 26;46(5):4333–47. doi: 10.1007/s11357-024-01138-8 (PMC11336001; doi:10.1007/s11357-024-01138-8)
Supplement: Supplementary file 1 — Supplementary file1 Supplementary Figure 1. Conosrt diagram for recruitment to the study. (DOC 27 KB) [file 11357_2024_1138_MOESM1_ESM.doc]

CONSORT diagram showing the flow of participants through each stage of the trail

Assessed for eligibility

(n = 87)

**Enrollment**

Excluded (n =4)

Not meeting inclusion criteria

(n = 4)

Refused to participate

(n =0)

#

**Allocation**

**Follow up**

**Analysis**

Recruited (n=83)

Allocated to intervention (n = 83)

Lost to follow up (n = 0)

Discontinued intervention (n = 3)

n=1 - withdrew as was vegetarian

n=1 - did not disclose a health condition that was an exclusion criteria

n=1 - was taken ill during the study and had to withdraw

Analysed all 12 week outcomes except epigenetic age

(n = 80)

Analysed for epigenetic age (n=79)

Excluded from analysis due to quality control of sample

(n =1)
