# Supplementary figures and images for: A combination nutritional supplement reduces DNA methylation age only in older adults with a raised epigenetic age
Source: GeroScience. 2024 Mar 26;46(5):4333–47. doi: 10.1007/s11357-024-01138-8 (PMC11336001; doi:10.1007/s11357-024-01138-8)

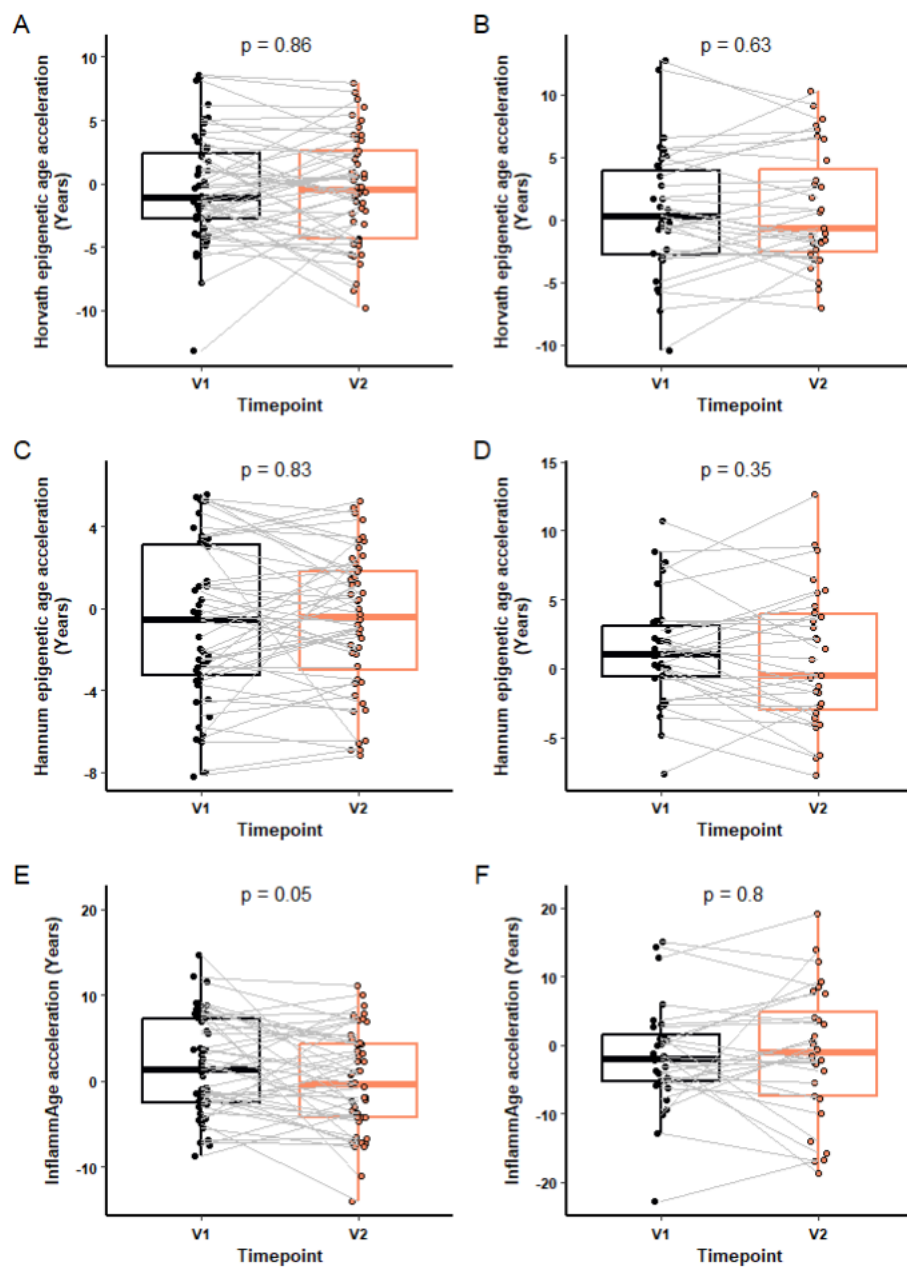

Supplement: Supplementary file 2 — Supplementary file2 Supplementary Fig 2: Effect of sex on epigenetic age response to supplement. The degree of epigenetic age acceleration in individuals at V1 and V2 were compared by the Horvath clock for females (A, n=48) and males (B, n=31), by the Hannum clock for females (C, n=48) and males (D, n=31), and by the InflammAge clock for females (E, n=47) and males (F, n=28). For each box plot the central line is median, the bottom line is the 1st quartile (Q1), the top is the 3rd quartile (Q3). The whiskers represent calculated minimum and maximum values using the interquartile range (IQR). P values are shown above each compared dataset. Statistical analysis was carried out using paired Student t-test. (PDF 165 KB) [file 11357_2024_1138_MOESM2_ESM.pdf]
