# Supplementary material for: A combination nutritional supplement reduces DNA methylation age only in older adults with a raised epigenetic age
Source: GeroScience. 2024 Mar 26;46(5):4333–47. doi: 10.1007/s11357-024-01138-8 (PMC11336001; doi:10.1007/s11357-024-01138-8)
Supplement: Supplementary file 3 — Supplementary file3 (DOCX 21 KB) [file 11357_2024_1138_MOESM3_ESM.docx]

**Supplementary Table 1 Participant Inclusion and Exclusion Criteria**

| **Inclusion criteria** | **Exclusion criteria** |
| --- | --- |
| - Age ≥60 years. - Ability to provide informed consent. - Willing to stop taking multivitamins, and dietary supplements containing vitamins C and D, B3, DHA/EPA, Olive polyphenols, resveratrol and astaxanthin or food/beverage products supplemented with the above ingredients 3 weeks prior to and throughout the study. - Able to travel to the clinic for initial and subsequent evaluations. | - Current smokers, or ex smokers who have stopped within the last 12 months, or are using nicotine replacement products. - History of diabetes, myocardial infarction, congestive heart failure, kidney failure, liver disease or stroke. - Untreated thyroid disorder, cancer, active neoplasms. - Untreated gastrointestinal, or pulmonary diseases. - Surgery or trauma in last 60 days. - Inflammatory diseases, autoimmune diseases or recent infection in last 60 days. - Allergies to any of the ingredients to be studied. - Currently taking or using multivitamins or dietary supplements containing vitamins C and D, B3, DHA/EPA, Olive polyphenols, resveratrol and astaxanthin or food/beverage products supplemented with the above ingredients. - Currently taking medication known to be metabolised by CYP3A. - Currently taking any of the following medications, which confound the effects of inflammation: Tamoxifen, Cyclosporine A, immunosuppressants or Anti TNF inhibitors, NSAIDs. - Currently taking any of the following anticoagulant drugs (blood thinners): Warfarin, Direct oral anticoagulant drugs (DOACs), and antiplatelet drugs including aspirin. - At risk of bleeding complications, including those with inherited bleeding disorders (such as haemophilia), or increased risk hemorrhagic stroke or events. - Unable or unwilling to maintain current lifestyle throughout study such as eating habits, exercise habits, etc. - Assessed as being frail by Fried phenotype criteria. |
